# Erratum to: Mechanistic attributes of S100A7 (psoriasin) in resistance of anoikis resulting tumor progression in squamous cell carcinoma of the oral cavity

**DOI:** 10.1186/s12935-015-0244-7

**Published:** 2015-10-08

**Authors:** Kaushik Kumar Dey, Siddik Sarkar, Ipsita Pal, Subhasis Das, Goutam Dey, Rashmi Bharti, Payel Banik, Jay Gopal Ray, Sukumar Maity, Indranil kulavi, Mahitosh Mandal

**Affiliations:** School of Medical Science and Technology, Indian Institute of Technology, Kharagpur, 721302 West Bengal India; Dr Rafi Ahmed Dental College and Hospital, Kolkata, 700014 West Bengal India; Calcutta Medical College, Kolkata, 700073 West Bengal India; Bankura Sammilani Medical College, Bankura, 722101 West Bengal India

## Erratum to: Cancer Cell Int (2015) 15:74 DOI 10.1186/s12935-015-0226-9

Unfortunately, the original version of this article contained an error. The name of one author Jay Gopal Ray was included incorrectly. The correct name can be found below:

Jay Gopal Ray

